# Adenomatous Polyposis Coli (APC) Promoter Gene Methylation in Urine-Derived DNA: A Non-invasive Biomarker for Early Bladder Cancer Detection and Tumor Aggressiveness

**DOI:** 10.7759/cureus.72055

**Published:** 2024-10-21

**Authors:** Mouna Aqerrout, Imane Mharrach, Kaoutar Anouar Tadlaoui, Abdelilah Laraqui, Mohamed Rida Tagajdid, Khalid Ennibi, Moulay Mustapha Ennaji

**Affiliations:** 1 Laboratory of Virology, Oncology, Biosciences, Environment and New Energies, Faculty of Sciences and Techniques Mohammedia, University Hassan II of Casablanca, Mohammedia, MAR; 2 Center of Virology, Infectious and Tropical Diseases, Mohammed V Military Teaching Hospital, Rabat, MAR; 3 Faculty of Medicine and Pharmacy, Mohammed V University, Rabat, MAR; 4 Sequencing Unit, Laboratory of Virology, Royal School of Military Health Service, Rabat, MAR

**Keywords:** apc promoter methylation, bladder cancer biomarker, epigenetic alterations, non-invasive diagnosis, urine-derived dna

## Abstract

Background

Bladder urothelial carcinoma (BLCA) is a major cause of morbidity and mortality worldwide, largely due to the high frequency of disease relapse and the lack of efficient endoscopic diagnostic methods. This study aimed to address this clinical gap by evaluating the potential of using adenomatous polyposis coli (APC) gene promoter methylation as a biomarker detectable in urine DNA of individuals with BLCA.

Methods

Methylation-specific PCR was used to determine the methylation status of the APC promoter gene in 50 bladder carcinoma patients and 50 apparently healthy individuals. Electrophoresis on agarose gel was performed for the detection of PCR products. Statistical analysis was conducted using Excel, SPSS, and Python to assess correlations and significance.

Results

APC promoter methylation was detected in 34 (68%) of bladder cancer cases but in only eight (16%) of healthy controls, indicating a strong association between APC promoter methylation and bladder cancer (p < 0.001). High-grade tumors were found to have significantly higher levels of APC promoter methylation, suggesting a link between APC methylation and tumor aggressiveness (p = 0.048). Smoking was identified as a significant risk factor for BLCA (p < 0.001), but no correlation was observed with the tumor stage.

Conclusion

APC promoter gene methylation shows a diagnostic value for BLCA and may be useful as a non-invasive marker for early detection. This study highlights the clinical utility of using a simple urine test to detect bladder cancer, particularly in early stages, and suggests that combining APC methylation with other specific biomarkers could enhance diagnostic accuracy.

## Introduction

It has been reported by GLOBOCAN estimates in 2018 that bladder urothelial carcinoma (BLCA) is the 10th most common cancer worldwide and statistics have estimated 549,000 new cancer cases and 200,000 death cases each year [1]. Bladder cancer has a high incidence and recurrence rate, prompting the need for new methods of early detection [2]. The current gold standard for diagnosis is cystoscopy, an invasive procedure involving biopsies that cause discomfort and incur high costs for both patients and healthcare systems [3,4]. Given the limitations of cystoscopy, non-invasive diagnostic techniques have become crucial, especially for detecting bladder cancer at an early stage [5].

DNA methylation, an epigenetic modification involving the addition of a methyl group to cytosine residues in Cytosine-phosphate-Guanine (CpG) islands, has emerged as a promising biomarker for non-invasive cancer detection [6]. Methylation in gene promoter regions can lead to gene silencing, contributing to cancer progression by affecting tumor suppressor genes [7]. Numerous studies suggest that aberrant DNA methylation patterns play a significant role in carcinogenesis and are associated with cancer initiation and progression, making DNA methylation an ideal candidate for non-invasive early cancer detection [8,9].

The study of epigenetic mutations in genes and especially oncogenes is a key path that can result in interesting discoveries about cancer in general. In our case, BLCA has been associated with numerous oncogenes; one of them is the adenomatous polyposis coli (APC) gene. The APC gene is placed in chromosome region 5q21-22 and is a tumor suppressor gene [10]. It has 15 exons and is crucial for cellular motility, proliferation, DNA repair, and many more cellular functions and interactions regulated by its APC protein [11]. Also, normal tissues typically do not include methylation in the CpG islands, which are small areas of genomic DNA found in the promoter region of genes. These areas may get methylated in specific illness conditions, such as when a tumor forms. There is growing evidence that aberrant DNA methylation plays a role in both the precancerous and malignant stages of human carcinogenesis [12]. Evidence from various reports has suggested the involvement of the APC gene in tumorigenesis of many cancer types, colorectal, gastric, and more [13]. For BLCA, the Wnt pathway antagonist gene is found to be one of the major tumor suppressor genes with altered sequences in the methylation patterns of BLCA cases compared to normal cases [14]. Consequently, the development of different human cancers is caused by the stimulation of the Wnt/β-catenin signaling pathway, which is triggered by the inactivation of Wnt-antagonist-related genes via promoter hypermethylation [15].

Urine-derived DNA represents a compelling source for non-invasive cancer detection due to its ease of collection and the presence of tumor DNA shed by bladder tumors into the urinary tract [16]. Methylation-specific PCR (MSP) for the APC promoter gene is a sensitive technique that can detect even low levels of methylation in specific genes, making it ideal for analyzing urine-derived DNA [17]. It has been demonstrated that it can also be a useful diagnostic and a potential biomarker for patients with BLCA [18,19].

This research aimed at establishing the potential of testing APC gene promoter methylation as a non-invasive BLCA biomarker for early diagnosis. Accordingly, the purpose of the present study was to delineate the correlation between the APC methylation status and other clinicopathological indicators in the management of BLCA since epigenetic modifications have recently been reported to play a part in tumorigenesis. We focused on a non-invasive approach, therefore using urine-derived DNA which can improve the diagnosis of BLCA and reduce invasiveness of the process. The current study contributes further to the persistent search for reliable biomarkers that may enhance early diagnosis, prognosis, and management of individual patients with BLCA.

## Materials and methods

Study design, collaboration, and duration

This study was conducted as a collaborative effort between authors based in different locations. We employed a combination of digital communication tools and regular meetings, to discuss progress, sharing documents, data, and resources securely and efficiently. This approach fostered continuous communication and enabled timely feedback, thus enhancing the overall coordination of the research efforts. The study commenced in February 2023 and was completed in September 2024, spanning a total duration of 19 months.

Patients and samples

A total of 100 participants were enrolled in this study, comprising 50 BLCA patients and 50 healthy control individuals. The BLCA patients were recruited from the military hospital of Rabat- Morocco, while the healthy controls were selected from individuals undergoing routine health check-ups with no history of cancer or other significant illnesses.

The inclusion criteria for the study consisted of BLCA patients with a histologically confirmed diagnosis of BLCA, aged between 40 and 80 years, who had not received prior chemotherapy or radiotherapy treatment. All participants provided written informed consent. Healthy controls were individuals with no history of malignancy or chronic illness, matched to the patient group by age and sex, with written informed consent obtained as well.

The exclusion criteria for both groups included a history of other malignancies or chronic diseases, prior or concurrent treatment with chemotherapy, radiotherapy, or immunotherapy, and inadequate urine sample volume, defined as less than 50 mL.

Urine sample collection

Midstream urine samples (50 mL) were collected in sterile tubes from all participants enrolled in our study. Urine samples were centrifuged at 2,000 x g for 10 minutes at 4°C to pellet cellular material right after the collection and then stored at -80°C until DNA extraction.

DNA extraction and bisulfite conversion

DNA from both patients and healthy control was extracted from the stored urine samples using the PureLink™ Microbiome DNA Purification Kit (Thermo Fisher Scientific, Waltham, MA, USA) following the manufacturer’s detailed protocol to ensure successful extraction. The extraction kit contains a series of steps including cell lysis, binding of DNA to a silica membrane, and washing to remove impurities, each step of the extraction utilizes a specific buffer. Finally, is the elution step of the purified DNA obtained from the membrane. This method is designed to efficiently extract microbial and human DNA from complex biological fluids such as urine and ensures that the extracted DNA is contaminant-free and ready for further analysis. The concentration and purity of extracted DNA were assessed using a NanoDrop 2000 spectrophotometer (Thermo Scientific, Wilmington, DE, USA). Samples with an A260/A280 ratio between 1.8 and 2.0 were deemed suitable for further analysis.

We employed the EpiJET Bisulfite Conversion Kit (Thermo Fisher Scientific, Vilnius, Lithuania) for bisulfite conversion carefully following the manufacturer’s protocol to ensure reproducibility. A total of 500 ng of genomic DNA was subjected to bisulfite treatment, a very important step in which unmethylated cytosines are converted to uracil, while methylated cytosines remain for the ability to differentiate between methylated and unmethylated regions. The reaction was carried out with an initial denaturation at 98°C for 10 minutes to ensure complete conversion, then a prolonged incubation at 64°C for 2.5 hours to drive the chemical reaction. Lastly, the DNA was purified and finally eluted in 20 µL of elution buffer. After the conversion, DNA was stored at -20°c until usage.

Methylation-specific PCR (MSP)

MSP was employed to assess the methylation status of the APC promoter A1 gene post-bisulfite conversion of our urine-derived DNA samples. We used two sets of primers to specifically detect methylated and unmethylated sequences [20]. For the unmethylated form (APC-U), the forward primer used was 5’-GTGTTTTATTGTGGAGTGTGGGTT-3’ and the reverse primer was 5’-CCAATCAACAAACTCCCAACAA-3’. For the methylated form (APC-M), the forward primer used was 5’-TATTGCGGAGTGCGGGTC-3’ and the reverse primer was 5’-TCGACGAACTCCCGACGA-3’. Each MSP reaction was conducted in a final volume of 25 µL containing a mixture of 1x PCR buffer, 0.2 mM of each dNTP, 500 ng of each primer, 0.2 U of Taq PCR Master mix (Vazyme Biotech, China), and 50 ng of the modified DNA. Tubes containing the PCR mixture underwent a hot start at 95°C for 5 minutes to ensure enzyme activation. The amplification was carried out in a PerkinElmer 2400 Thermal Cycler® (Scientific Support, Inc., Hayward, CA) for a total of 35 cycles under the following conditions: denaturation at 95°C for 30 seconds, annealing at 60°C for 30 seconds, and extension at 72°C for 30 seconds. A final extension step at 72°C for 10 minutes was included to complete the amplification. The MSP products were then employed in further analysis on a 1.5% agarose gel stained with ethidium bromide and then visualized under UV light to assess our results with the presence or absence of bands signaling methylation or non-methylation of our DNA samples for APC promoter gene which were compared to DNA ladder (Thermo Scientific).

Statistical analysis

Comparisons of methylation levels between BLCA patients and controls were conducted using the chi-square test. The correlation between clinicopathological parameters and methylation status of the patients was carried out using the chi-square test (or Fisher's Exact Test) and Mann-Whitney U Test, the threshold for statistical significance was set at p < 0.05. The diagnostic performance of APC promoter methylation was assessed using the area under the ROC curve (AUC). All statistical analyses were performed using Excel (Microsoft Corporation, Redmond, USA), IBM SPSS Statistics for Windows, Version 1 (Released 2001; IBM Corp., Armonk, New York, United States), and Python's Matplotlib/Seaborn/Scikit-learn libraries (Python version 3.11.0).

## Results

The study we conducted to evaluate the potential biomarker role of the APC promoter gene of BLCA diagnosis recruited 100 individuals, from which 50 were BLCA patients and the other half were healthy people as controls. The clinicopathological data of patients and controls are represented in Table 1. Out of 50 (50%) BLCA patients, the sex distribution was male dominated by 39 (78%) men and 11 (22%) women, and the 50 (50%) healthy controls. The tumor stage was distributed showcasing early cancer stages pTa /pT1 with 37 (74%) cases and (non-invasive tumors) and 13 (26%) cases with advanced stages (≥ pT2 (invasive tumors). Low BLCA grade was found in 29 (58%) patients and high grade in 21 (42%) patients. Samples of urine from 50 BLCA patients and 50 healthy controls were employed to extract DNA, which was then processed using a bisulfite reagent and utilized as a template for methylation-specific PCR analysis utilizing pairs of gene-specific primer sets.

**Table 1:** Clinicopathological parameters of BLCA patients. *: Significant p-value BMI: Body Mass Index; BLCA: Bladder Urothelial Carcinoma

Tumor features	BLCA group N = 50 (50%)	Control group N = 50 (50%)	p-value	χ² Value
Sex ratio				
Male	39 (78%)	34 (68%)	0.368	0.81
Female	11 (22%)	16 (32%)		
Smoking status				
Smokers	38 (76%)	18 (36%)	<0.001*	14.65
Non-smokers	12 (24%)	32 (64%)		
Family history of bladder cancer				
Positive	20 (40%)	N/A	N/A	N/A
Negative	30 (60%)	N/A		
Age at diagnosis (years)				
<60	14 (28%)	13 (26%)	1.0	0.00
>=60	36 (72%)	37 (74%)		
Medical background				
Yes	32 (64%)	N/A	N/A	N/A
No	27 (54%)	N/A		
Surgical history				
Yes	19 (38%)	N/A	N/A	N/A
No	31 (62%)	N/A		
BMI (kg/m²)				
<20	2 (4%)	0 (0%)	0.314	2.32
>=20 <25	30 (60%)	34 (68%)		
>=25	18 (36%)	22 (44%)		
Pathological T-stage				
pTa	19 (38%)	N/A	N/A	N/A
pT1	18 (36%)	N/A		
pT2	10 (20%)	N/A		
pT3, pT4	3 (6%)	N/A		
Tumor grade at diagnosis				
High grade	21 (42%)	N/A	N/A	N/A
Low grade	29 (58%)	N/A		
Muscle Invasion				
No	39 (78%)	N/A	N/A	N/A
Yes	11 (22%)	N/A		

**Figure 1:**
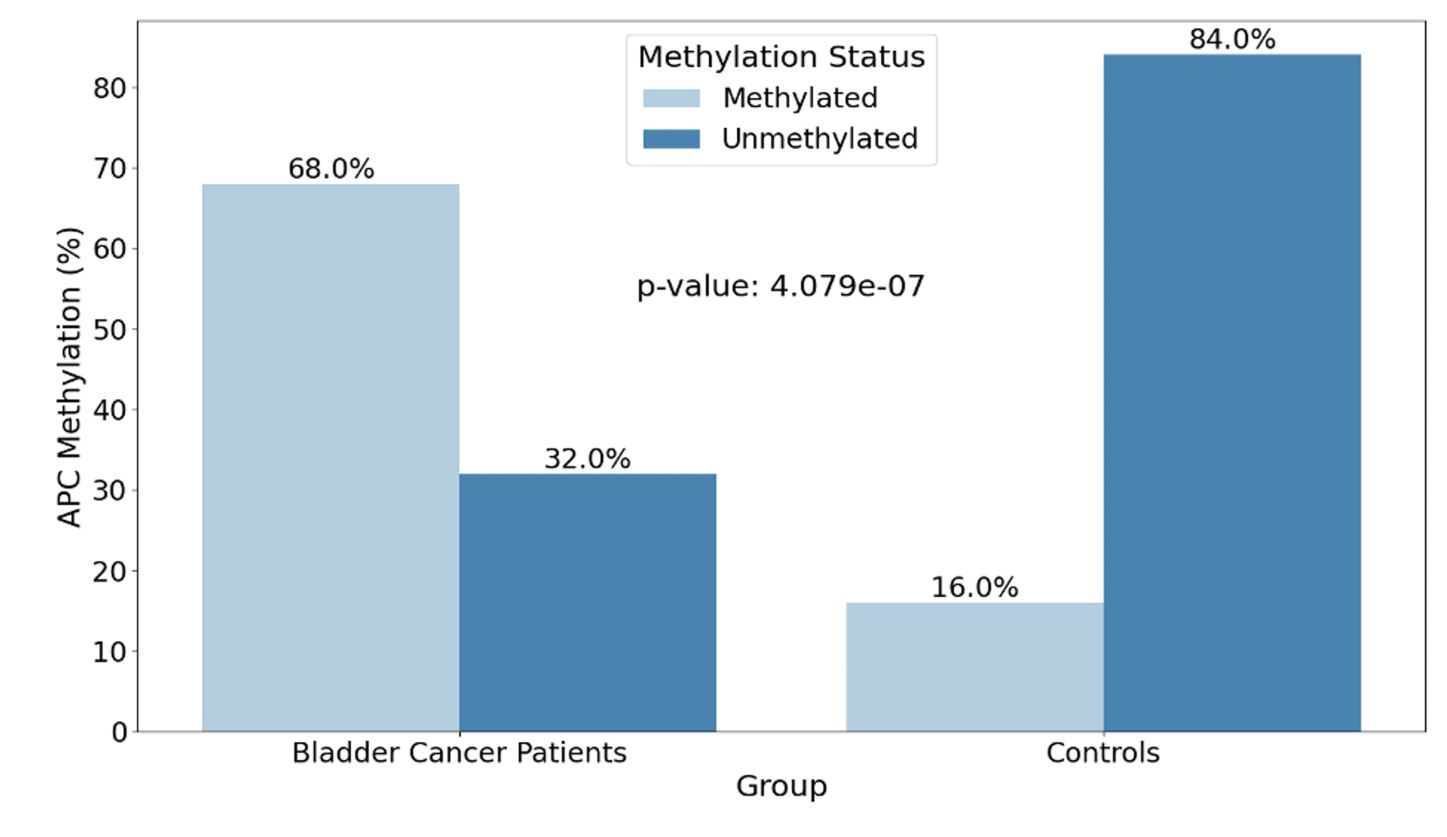
Comparison of the APC methylation status between BLCA patients and controls. This bar chart illustrates the percentage of APC promoter methylation in BLCA patients (68% methylated, 32% unmethylated) compared to controls (16% methylated, 84% unmethylated), with a significant p-value< 0.001 using the chi-square test. BLCA: Bladder Urothelial Carcinoma; APC: Adenomatous Polyposis Coli

The study’s results have revealed that among BLCA patients, 34 (68%) showed APC promoter gene methylation, while for healthy controls, only 8 (16%) exhibited methylation. Conversely, for healthy controls, non-methylation was more dominant with 42 (84%) compared to BLCA patients with 16 (32%) showcasing non-methylation for the APC promoter gene (Figure 1). The bar chart representing these findings shows a significant difference between methylation status frequency in both groups, patients and controls (p-value <0.001).

APC methylation was found to be significantly associated with the tumor grade of BLCA patients (Table 2). Among patients with high-grade tumors, 18 (52.9%) exhibited methylation of the APC gene, while 16 (47.1%) of low-grade tumors demonstrated methylation. This difference was statistically significant with a p-value of 0.048, indicating that APC methylation is more prevalent in high-grade, aggressive BLCAs. This suggests that the methylation status of the APC gene may be linked to tumor aggressiveness, and APC methylation could potentially serve as a biomarker to identify high-risk BLCA patients.

**Table 2:** Correlation between the APC methylation status and clinicopathological parameters of BLCA patients. *: significant p-value BLCA: Bladder Urothelial Carcinoma; APC: Adenomatous Polyposis Coli

Clinicopathological parameters	Methylated cases N=34 (68%)	Unmethylated cases N=16 (32%)	p-value	χ² Value
Sex				
Male	27 (79.4%)	12 (75.0%)	0.725	0.12
Female	7 (20.6%)	4 (25.0%)		
Smoking status				
Smokers	30 (88.2%)	8 (50.0%)	< 0.001*	6.75
Non-smokers	4 (11.8%)	8 (50.0%)		
Tumor grade at diagnosis				
High	18 (52.9%)	3 (18.8%)	0.048*	3.91
Low	16 (47.1%)	13 (81.3%)		
Pathological T-stage				
pTa	11 (32.4%)	8 (50.0%)	0.112	5.99
pT1	11 (32.4%)	7 (43.8%)		
pT2	10 (29.4%)	0 (0.0%)		
pT3-4	2 (5.9%)	1 (6.3%)		

A highly significant association was observed between smoking status and APC methylation. Among BLCA patients with a history of smoking, 30 (88.2%) exhibited APC gene methylation, compared to four (11.8%) non-smokers. A p-value of <0.001 underscores a strong correlation between smoking and the likelihood of APC methylation in BLCA patients. This finding is in line with the established relationship between smoking and epigenetic alterations in cancer. Smoking is a known risk factor for BLCA, and the higher frequency of APC promoter methylation in smokers suggests that smoking may contribute to the epigenetic changes that drive bladder carcinogenesis.

In contrast, tumor stage and sex did not demonstrate statistically significant associations with APC promoter gene methylation (p-values > 0.05). Although these parameters are important in the clinical characterization of BLCA, their lack of significant correlation with APC methylation in our cohort suggests that the epigenetic changes associated with APC may be more closely related to the aggressiveness of the tumor (as indicated by grade) and environmental or lifestyle factors such as smoking, rather than the stage at diagnosis or demographic factors.

**Figure 2:**
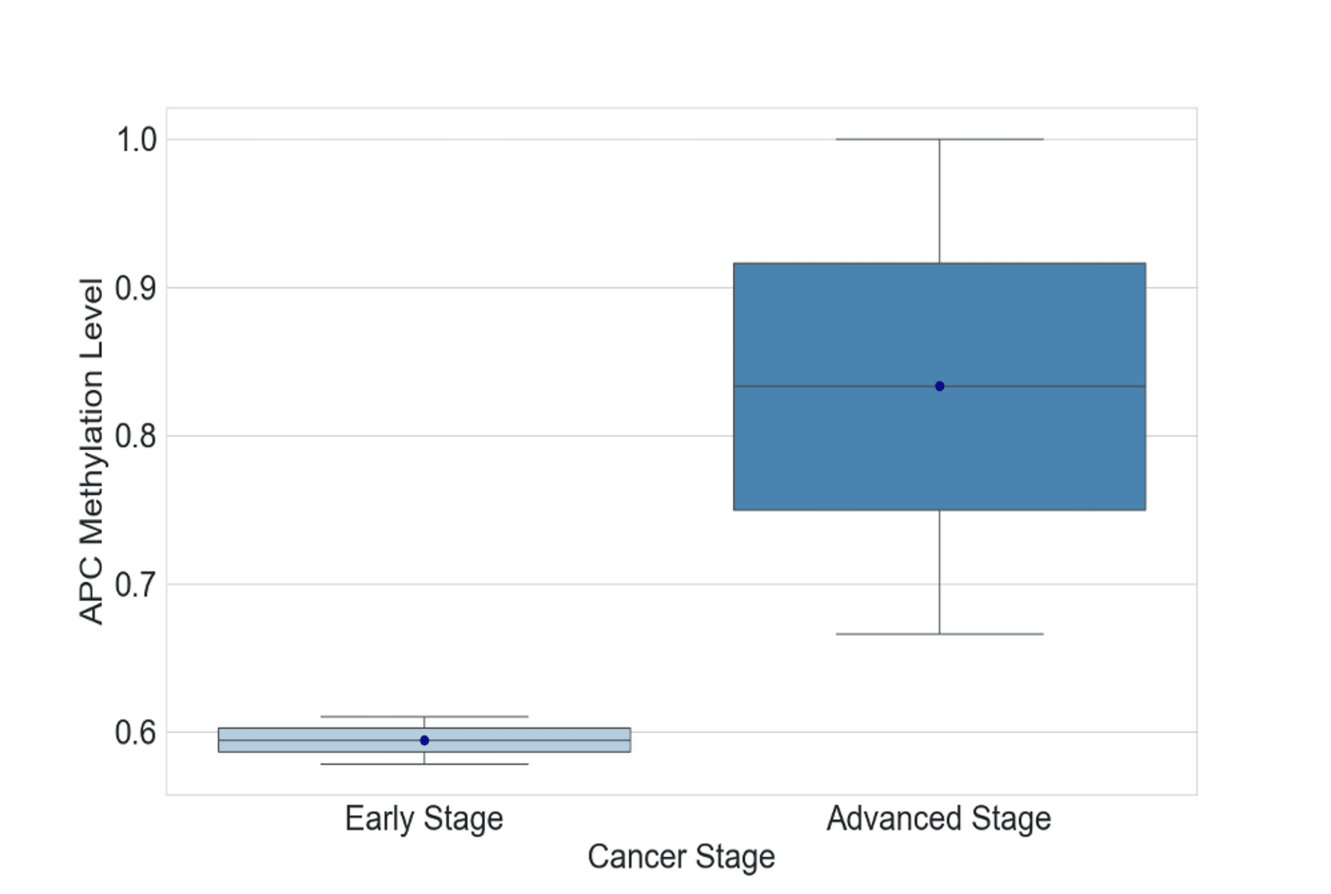
APC methylation levels in early and advanced BLCA stages. This box plot shows the distribution of APC methylation levels in BLCA patients, comparing early-stage (low methylation) and advanced-stage (high methylation) groups. The advanced stage exhibits a wider range and higher methylation levels compared to the early stage. A p-value of 0.112 was calculated using the Mann-Whitney U test. BLCA: Bladder Urothelial Carcinoma; APC: Adenomatous Polyposis Coli

A comparison of APC gene methylation levels between early and advanced tumor stages was visualized using a box plot (Figure 2). The analysis grouped tumor stages into early stages (pTa and pT1) and advanced stages (pT2 and pT3-pT4). The mean methylation level was calculated for each group, showing a trend toward higher methylation in the advanced stages. However, while there was a visible difference between the two groups, statistical analysis using the chi-square test revealed that the difference was not statistically significant (p = 0.112). This result suggests that although there may be a tendency for higher methylation in more advanced stages of BLCA, the observed differences in our dataset did not reach statistical significance.

**Figure 3:**
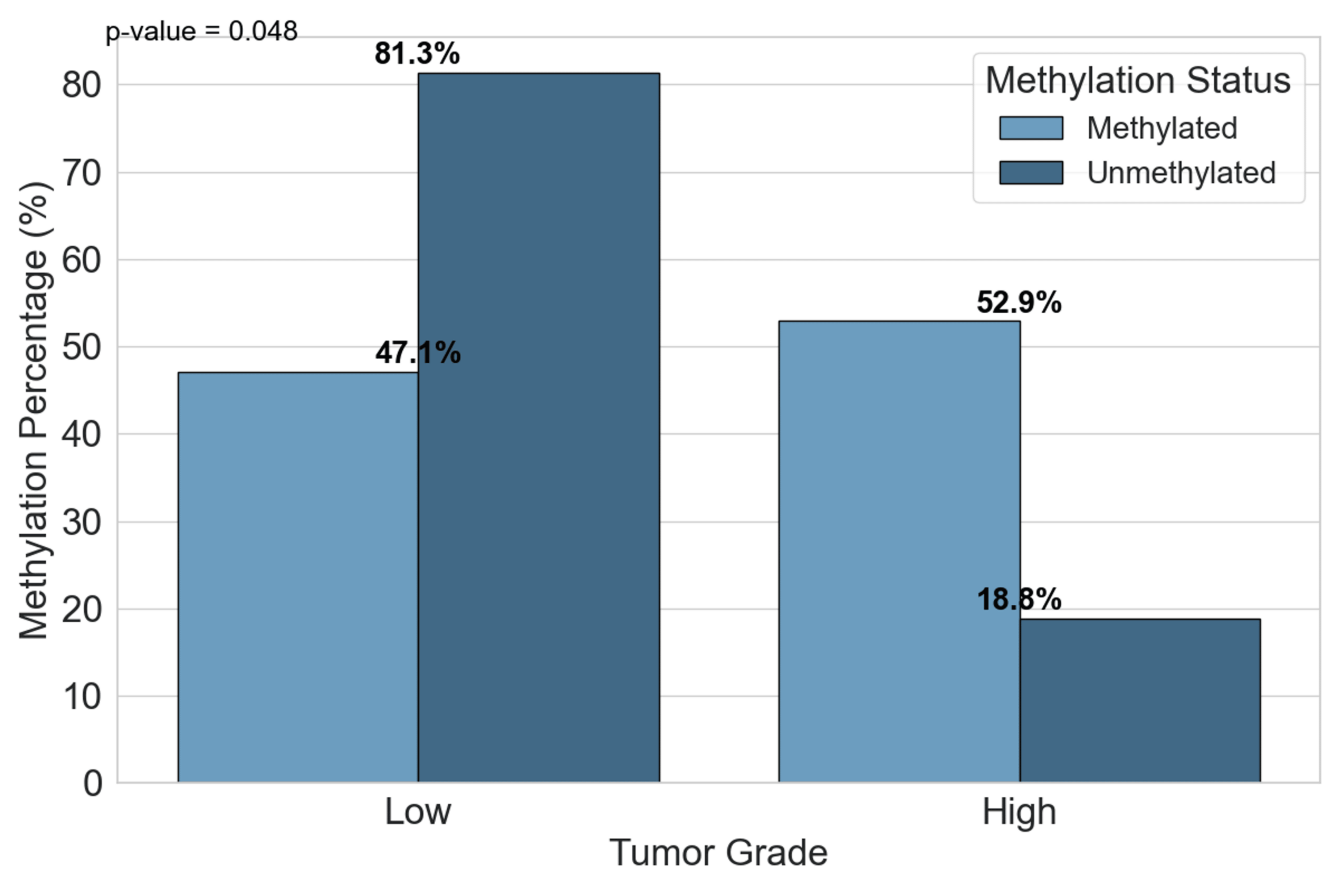
Methylation status distribution in terms of the tumor grade of BLCA patients. The bar chart shows the methylation status of different tumor grades. The bar chart indicates that high-grade tumors are more likely to be methylated than low-grade tumors. The p-value calculated with the chi-square test suggests that this difference in methylation status is statistically significant. BLCA: Bladder Urothelial Carcinoma

The distribution of APC methylation status between tumor grades is presented in Figure 3. Among the low-grade BLCA patients, 16 (47.1%) showed methylation, while 13 (81.3%) were unmethylated. In contrast, high-grade patients exhibited a slightly higher methylation rate, with 18 (52.9%) methylated and only 3 (18.8%) unmethylated. The observed difference in the methylation status between low and high tumor grades was statistically significant (p = 0.048), suggesting a potential association between increased APC methylation and tumor progression in BLCA.

**Figure 4:**
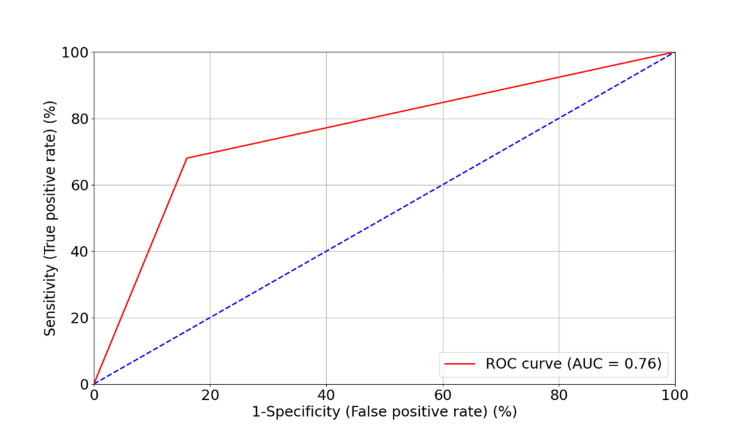
ROC curve for APC gene methylation as a diagnostic biomarker for BLCA. The ROC curve illustrates the diagnostic performance of APC promoter methylation status in distinguishing between BLCA patients and healthy controls, with an area under the curve (AUC) of 0.76, indicating a moderate discrimination ability. BLCA: Bladder Urothelial Carcinoma; APC: Adenomatous Polyposis Coli

APC promoter gene methylation showed a good diagnostic performance in differentiating BLCA patients from healthy controls. The sensitivity of 68% indicates that it correctly identifies 68% of the cancer cases, while the specificity of 84% suggests a strong ability to correctly classify healthy individuals. The diagnostic performance of APC gene methylation as a biomarker for BLCA was assessed using a receiver operating characteristic (ROC) curve (Figure 4). The ROC curve demonstrated an area under the curve (AUC) of 0.76, indicating a moderate discriminatory power of APC methylation status in distinguishing BLCA patients from healthy controls. The optimal cut-off point, based on the Youden index, yielded a sensitivity of 68% and a specificity of 84%, suggesting that APC gene methylation has a high potential as a diagnostic marker for BLCA. The 95% confidence interval (CI) for the AUC was [0.66-0.86], and the p-value was < 0.001, confirming the statistical significance of the APC methylation status as a diagnostic marker.

## Discussion

In our study, the main aim was to unravel the potential of the APC promoter as a biomarker for BLCA using urine-derived DNA as a non-invasive approach to conquer this disease. As showcased in multiple reports, the methylation status of a gene is an indicator of carcinogenesis or at least tumor formation. Thus, the methylation status of the APC gene and its veritable link to BLCA has been the focus of many studies in the past years [21,22]. However, the diagnostic value of the APC promoter gene is not well documented yet and its relationship with BC is still mystical, especially when it comes to the effects of APC hypermethylation on cells and protein expression. APC gene, being a well-known characterized tumor suppressor gene, when it comes to hypermethylation, this gene was proven to be associated with different types of cancer, colorectal, breast, etc., and its significative correlation with tumor characteristics and cancer cell formation. It is inevitable to turn a blind eye to the diagnostic value and the involvement of methylation status of APC promoter as a biomarker for cancer detection. Although several studies have showcased the role of the APC promoter gene as an effective diagnostic marker, in the case of BLCA it is poorly investigated and the increasing prevalence rate of this type of cancer each year is alarming, hence, the need for non-invasive diagnostic methods for BLCA detection. We conducted this study in the hope that through APC promoter methylation event, we can dive into a novel approach and investigate the potential of APC promoter methylation as a biomarker in bladder carcinogenesis.

So, we evaluated the significant difference in the percentage of methylation status between the two groups enrolled in our study, the patients and the control group, and among the BLCA patients, 34 (68%) showcased methylation of the APC promoter gene; meanwhile, eight (16%) of the healthy control group showed promoter methylation. This difference was met by a p-value <0.001, signaling that the difference between the APC promoter methylation between the two groups is statistically significant. Also, urinary APC promoter gene methylation was grade but not stage-dependent, indicating that their usefulness is limited to high-grade tumors but of course, this statement needs to be confirmed throughout further studies. According to our research, at the early stages of the tumor, methylation was moderately prevalent but it was found to be increasing in more advanced stages mostly in pT2 where all samples were methylated but no significant association between tumor stage and the methylation status of APC promoter was observed (p-value = 0.112). On the contrary, for tumor grade, in low-grade tumors, the distribution was even between methylation and unmethylation of the APC promoter with 16 (47.1%) and 13 (81.3%) respectively, but in the case of high-grade tumors, methylation was a little higher occurring in 18 (52.9%) of cases and with a p-value of 0. 048 < 0.05 indicating a significant correlation between APC gene promoter methylation and the clinicopathological parameter tumor grade. Overall, these findings suggest that methylation is more prevalent in higher grades and advanced stages of the tumor. Our results were in line with many reports investigating DNA methylation-based biomarkers in BLCA, and more specifically investigating the same aspect of the methylation of APC gene promoter. The investigation carried out by Bilgrami et al. [23] on various tumor suppressor genes evaluated the correlation between their promoter methylation with tumor grade and BLCA invasiveness; this study found significant hypermethylation of the APC gene promoter in the 33 high-grade BLCA patients studied compared to the 43 that were low grade (p-value <0.001) and normal controls (p-value<0.05). The same deduction was made by Berrada et al. on APC promoter methylation, showing that it presented an aberrant methylation regardless of clinicopathological features of BLCA patients and its frequency was 100% in all the cases studied, suggesting a very high biomarker value for the diagnosis of BLCA in its early stages since the hypermethylation was observed in all stages and grades of cancer [24]. Moreover, this report showcases the methylation status of this gene in both urine and tissue samples, with the frequency of the methylation in urine being 23 (79.3%) and the specificity of methylation detection in urine sediments reaching 100%. Previous reports have highlighted the role of methylation of the promoter region [25], suggesting that it can lead to mutation or gene deletion with the loss of the function of the tumor suppressor gene. This epigenetic change that occurs in the promoter region of the APC gene is suggested to have one of, if not the highest methylation rate out of a panel of genes studied by many reports [26,27]. Thus, methylation levels detection of APC promoter from urine can be a great and handy non-invasive method for BLCA detection [28]. However, there are very few contradicting reports [18] and [29] that found low methylation frequencies when studying the APC promoter gene and associated it with poor prognostic in urothelial cancer and opened the door to the organ-specific gene promoter panels and their importance in epigenetic alteration studies, to ensure the reliability and trust the outcome of the diagnostic biomarker approach utilized. In addition to tumor grade, smoking has established a strong reputation for being a major environmental and lifestyle risk factor for cancer in general. Its involvement and relevance in BLCA specifically have gained a lot of attention over the last few years, mainly in association with genetic alterations that can eventually lead to this disease. As DNA methylation gained a lot of attention for being considered a potential non-invasive biomarker for BLCA, it has been demonstrated by numerous reports that DNA aberrant methylation is not only present in BLCA individuals but its risk increases in smokers [30], which highlights our findings in terms of the correlation between smoking status and APC promoter gene methylation (p-value <0.001). Substances present in cigarette smoke may induce such epigenetic modifications [31], which is why methylation of tumor suppressor genes, like APC, can serve as early indicators of malignant transformation [32].

On the subject of predictive models to evaluate the diagnostic power of the APC promoter in bladder carcinogenesis, the ROC curve analysis demonstrated that our model has great ability to distinguish between BLCA patients from healthy controls which indicates that the methylation status of APC promoter serves as a promising biomarker for BLCA with an AUC of 0.76. The sensitivity and specificity values, 68% and 84% respectively at the optimal threshold of the curve are also great indicators that APC promoter methylation is a great diagnostic tool for BLCA, on the one hand, the sensitivity offers a crucial clinical insight which is the correct identification of BLCA cases. On the other hand, specificity reduces the false positive rate by ensuring a good identification of cancer-free individuals. Our model showcased a high significance (p-value<0.001) which solidifies the association between the methylation status of the APC promoter gene and its role as a biomarker and as a potentially non-invasive diagnostic procedure for BLCA. This aligns with major studies that provided important research and significant evidence on the association between APC promoter aberrant methylation and the risk of BLCA and affirmed the potential of methylated urine APC promoter as a reliable biomarker for bladder carcinogenesis [22]. Methylated biomarkers have shown great accuracy according to research in recent years, working with urine-derived DNA for epigenetic alterations, for methylation studies has more advantages than working on tissue samples [33], since the ROC curves show greater sensitivity, specificity, and superior AUC when it comes to APC promoter gene marker derived from urine DNA. In fact, methylation markers in general tend to exhibit a more powerful diagnostic and an overall great accuracy, that surpasses 65% in global accuracies [34]. One of the major factors of early tumorigenesis is known by many reports to occur due to hypermethylation of CpG island promoter, in our case, the APC promoter, so this insinuates that aberrant methylation doesn’t translate to invasive BLCA [35,36], which justifies even further the possibility for APC promoter methylation in urine samples to be an ideal early detection biomarker and a non-invasive diagnostic approach for BLCA. Several studies have demonstrated that methylation of CpG sites in urine can serve as promising markers for detecting or monitoring BLCA, and while APC gene methylation status is a promising biomarker, it might be more effective when used in combination with other biomarkers or diagnostic methods to improve overall predictive accuracy as suggested by many reports [16].

Our study highlights the potential of APC gene methylation as a promising non-invasive biomarker for the early detection of BLCA. The significant association between the APC methylation status and BLCA, combined with the robust diagnostic performance indicated by the ROC curve analysis, underscores the value of incorporating this biomarker into clinical practice. While the findings about APC promoter gene methylation as a non-invasive biomarker for BLCA are promising, there are some limitations. First, the sample size of 50 BLCA patients and 50 controls is relatively small, which might affect how well the results apply to a larger population. Also, we used MSP to assess methylation, but more advanced methods like qPCR or next-generation sequencing might give more accurate results and give us more informative and quantitative insights on the APC promoter gene expression in BLCA urine samples. The study only focused on APC gene methylation and didn’t look at other potential markers that could improve the diagnostic process. Lastly, because the study is cross-sectional, we can’t establish a direct cause-and-effect relationship between APC methylation and cancer progression. While these findings are encouraging, further validation in larger, diverse cohorts is necessary to confirm the generalizability of these results. Future research should also explore the integration of APC methylation with other molecular biomarkers to enhance diagnostic accuracy and develop a comprehensive panel for BLCA screening. Such efforts could lead to more personalized and effective approaches to cancer detection and management.

## Conclusions

In this work, we have demonstrated the importance of using urine-derived DNA and APC gene promoter methylation as a promising non-invasive biomarker in diagnosing BLCA. These findings should further the use of APC gene DNA methylation and DNA methylation in general as an effective diagnostic aid that does not require an invasive procedure as the ones used at the moment. Besides, this research states two significant facts: the dramatic role of epigenetic changes in the development of BLCA and the imperative to create novel and more tolerable diagnostic tools.

By following strict methods and proving a significant relationship between methylation profiles and clinicopathological features, we have provided fresh evidence in support of the perfect biomarker role of DNA methylation. This work provides the basis for subsequent investigations that will seek to confirm and enhance methylation-based diagnostic testing as a method of early detection that may lead to better management of this condition. We believe that by relying on non-invasive methods, the result of our work has the potential to provide a solid foundation for the development of less invasive cancer diagnostic tools, not only improving the quality of life of patients and cutting the expenses of healthcare services. Since the complications of BLCA are still very much present, especially the recurrence and progression, the demand for more accurate biomarkers is even higher. It is toward the goal of improving early diagnosis and differential treatment approaches consistent with the cancer type that this study contributes.
